# Less Citation, Less Dissemination: The Case of French Psychoanalysis

**DOI:** 10.3389/fpsyg.2016.01729

**Published:** 2016-11-03

**Authors:** Rémy Potier, Olivier Putois, Charlotte Dolez, Elliot Jurist

**Affiliations:** ^1^Department of Psychoanalytic Studies, Center for Research in Psychoanalysis, Medicine and Society, Paris Diderot University, Sorbonne Paris CitéParis, France; ^2^Psychoanalytic Psychopathology, Department of Psychology, Strasbourg UniversityStrasbourg, France; ^3^Political Sociology, Université Catholique de LouvainLeuven, Belgium; ^4^Doctoral Program in Clinical Psychology, City College of New York, City University of New YorkNew York, NY, USA

**Keywords:** digital humanities, globalization, French psychoanalysis, Anglo-American psychoanalysis, citation rate, national heritage, visibility, acknowledgment

## Introduction

The future of all publishing is open to question, and this is especially true in the case of psychoanalytic publishing. Stepansky (2009) has explored the future of psychoanalytic publishing with a particular emphasis upon how the digital era has had an impact upon the decline of scholarly publication in the United States. If this trend continues, the survival of contemporary psychoanalytic research will depend upon its capacity to embrace and utilize digital publishing.

Echoing this perspective, we tried to determine whether the seemingly small international visibility of contemporary French psychoanalytic research could be related to its lack of acknowledgment of the impact of digitalization on rules of writing and publishing.

We believe that achieving visibility doesn't chiefly depend on overcoming a language barrier (not such an issue for younger generations), cultural differences, or geographical distance (made irrelevant by the Internet). Rather, our intuition is that French psychoanalytic work would become more visible if it demonstrated familiarity with psychoanalytic work in English, citing it and engaging in dialogue.

Anglo-American psychoanalytic publications follow a specific rule of digitalized, database-anchored research. This rule is that the bibliometric value of a journal, which largely conditions its academic visibility and value, depends on the algorithmic measure of the number and type of cites that it receives from published articles published. (This is the case, in different ways, with the commonest algorithms: Thomson's Impact Factor and SCImago's Journal and Country Rank.) In other words, the algorithmic measure of this value ultimately depends on whether and how researchers cite a given journal—these citation practices will then, in turn, make it a more desirable publishing venue. The bottom line is that citations have a real effect, as they directly contributing to the space of academic publishing by differentiating journals both numerically and hierarchically.

It is plausible to imagine that an increase in the number of Anglo-American journal citations in French journals would be likely to produce a rise in international visibility for French journals. Of course, this is speculative and cannot (yet) be put to test. Let us begin with a preliminary effort: to draw on an original database in order (a) to determine whether French psychoanalytic productions are visible internationally, and (b) to clarify the citation practices comparatively between the French and Anglo-American contexts. To the best of our knowledge, this issue has not been addressed so far; in providing preliminary data and reflections, our hope is modest: to inspire discussion and debate.

To that end, we first measure the citational visibility of French vs. Anglo-American psychoanalytic journals across all disciplines and languages (their respective global outreach); we then relate this outreach measure to a geographic breakdown of psychoanalytic article citations, in order to make sense of specific geographical differences in citation practices.

## Methods

One of us (RP) put together a dedicated database, which is the first result of this research. This database draws on the data available in Elsevier's *Scopus* database, which indexes the highest number of Social Science and Humanities journals.

The first step was to establish a corpus of psychoanalytic journals, selected through content analysis. All of these journals refer to psychoanalysis either exclusively or significantly (by publishing psychoanalysis-oriented papers relevant to the fields of psychiatry and/or psychology). They have similar editorial processes and publishing circuits (e.g. non-open access); and their language is that of the publishing body's country (they are not bilingual). Being indexed in Scopus, they all have an SJR metrics.

The list of selected journals is the following:

1. 13 French journals: Annales Médico-Psychologiques, Cliniques Méditerranéennes, Enfances et Psy, Essaim, Evolution Psychiatrique, Figures de la Psychanalyse, Imaginaire et Inconscient, Information Psychiatrique, Psychiatrie de l'Enfant, Revue de Psychothérapie Psychanalytique de Groupe, Revue Française de Psychanalyse, Savoirs et Clinique, Topique.
2. 20 Anglo-American Journals: American Imago, American Journal of Psychoanalysis, British Journal of Psychotherapy, Contemporary Psychoanalysis, International Forum of Psychoanalysis, International Journal of Applied Psychoanalytic Studies, Journal of the American Psychoanalytic Association, Journal of Analytical Psychology, Journal of Child Psychotherapy, Psychoanalytic Dialogues, Psychoanalytic Inquiry, Psychoanalytic Perspectives, Psychoanalytic Psychology, Psychoanalytic Psychotherapy, Psychoanalytic Quarterly, Psychoanalytic Review, Psychoanalytic Social Work, Psychodynamic Practice, Psychoanalytic Study of the Child, Psychodynamic Psychiatry.

RP has collected the whole bibliometric data of this corpus of 33 journals (13 + 20) over the last 15 publication years (2000–2015).

The database variables are, for each journal over this period: SJR per year, number of article citations per year, number of cited articles, number of published citable articles per year, number of non-cited articles per year, country of origin of cites.

For Table 1, we started from the number of documents, the number of citations and the number of non-cited documents, and just calculated the percentage.

**Table 1:** **Ratio of citation and non-citation per psychoanalytic article depending on its geographic origin**.

**Cites/documents ratio, 2000-2015**	**Total of cites**	**Total of documents**	**Ratio**
French journals	12935	11370	1,14
Anglo-American journals	127727	12540	10,6
**Percentage of non-cited documents by origin, 2000-2015**	**Total of non-cited documents**	**Total of documents**	**Ratio**
French journals	8018	11370	70,52
Anglo-American journals	5342	12540	41,995

For Table 2, starting from the number of articles that cite each journal (with their geographic origin), we added the numbers and then calculated the percentage.

**Table 2:** **Breakdown of article citation ratios in French vs. Anglo-American journals of all disciplines**.

**Percentage of cites by origin, 2000–2015**	**Total of articles cites**	**Cited by French journals**	**Cited by Anglo-American journals**
French journals	6812	66,23% (*n* = 4512)	5,66% (*n* = 386)
Anglo-American journals	28204	1,20% (*n* = 339)	63,08% (*n* = 17791)

## Results

All of the following results are to be understood over a 15-year period (2000–2015).

### Global outreach of French vs. Anglo-American psychoanalytic articles

Are French psychoanalytic journals almost absent from the international research scene? A first step toward answering this question is to assess the global outreach of French vs. Anglo-American articles, by determining the ratio of citations per psychoanalytic article depending on its geographic origin (French vs. Anglo-American).

Aiming at a global outreach measure, we didn't differentiate between origin (French vs. Anglo-American vs. other) or disciplinary affiliation of citing journals.

For this Table 2 cites referring to one and the same article in the same document count as 2; and self-cites are included.

The results are telling: across all publication supports and languages, each French psychoanalytic article is cited about only once (1,14 %), while for each Anglo-American article, the ratio is of 10,6% - that is, about 10 times higher.

On the other hand, of the 11370 articles published in French psychoanalytic journals over the last 15 years, 70,52% haven't been cited in any support, while this figure drops to an average of 41,995% for the 12,540 articles published in Anglo-American journals. In other words, looking back today over the last 15 years, the global rate of non-citedness of Anglo-American psychoanalytic articles is almost twice as small as that of French articles.

### Geographic breakdown of psychoanalytic articles cites across all disciplines

We then sought to establish an overview of the geographic breakdown of psychoanalytic articles citations in French vs. Anglo-American journals of all disciplines.

In the following table, the unit isn't the citation, but the article: two citations of one and the same article count as 1, if the citation is in the same article, and as 2 if in two different articles.

Out of the 6812 total cites referring to articles published in French psychoanalytic journals, two thirds (66,23%) come from French journals (whatever their discipline), and only 5,66% from Anglo-American journals. On the other hand, out of the 28,204 total cites referring to articles published in Anglo-American psychoanalytic journals, 63,08% (17,791) come from Anglo-American journals, and 1,20% (339) from French Journals.

Thus comparatively, French psychoanalytic articles are cited almost 5 times as much (4,7 times) in Anglo-American journals (5,66%) as Anglo-American psychoanalytic articles in French journals (1,20%).

## Discussion

These data demonstrate that the global outreach of French psychoanalytic articles is 10 times smaller than that of Anglo-American ones (Table 1) and that, unsurprisingly, such articles are mostly referred to in French journals, wherein references to Anglo-American ones are almost nonexistent (Table 2).

### Limitations of the current data

One might argue that, since the considered time range is 2000–2015, the language barrier could partly account for the difference in outreach ratio, and for the virtual absence of Anglo-American citations in French journals—most of which are psychoanalytic. For in France, only recently has the language barrier genuinely ceased to become less of an issue. Nevertheless, the difference in outreach ratio is so striking, and the references to Anglo-American work in French journals so scarce, that it is doubtful whether language barrier could account for it.

Also, even though Scopus is the best available database for psychoanalytic journals, it doesn't—yet—index certain French psychoanalytic journals (such as *Research in Psychoanalysis* or *Adolescence*) which have quite an important readership amongst clinicians.

Most importantly, both outreach measures and geographic breakdown of citations are not, in the present article, restricted to psychoanalytic journals: they include all research and languages (Table 1). Indeed, when taking into account geographic breakdown (Table 2), they do not differentiate among the disciplines of the citing journals. Clearly, this would be important to examine in future studies.

We believe that, in light of the quantity of further database work required to reach such detail and accuracy and the absence of papers addressing the current topic, it makes sense to produce a few preliminary figures, in order to stimulate discussion and continuing research. For example, it would be important to look at outreach within different languages or disciplines; and specifically at the geographic breakdown of psychoanalytic citations.

The figures provided in the present paper constitute an intriguing proxy as to both the international outreach of French psychoanalytic research, and the specific citation practices at work in the French psychoanalytic community. French psychoanalytic work has quite a small outreach; and when cited, it is mostly in French journals—most of which are, most likely, psychoanalytic. Therefore, in view of the outreach ratio, it is likely that French psychoanalytic researchers (who arguably constitute most of the readership of French psychoanalytic work) do not cite their peers very often. And since we know (Table 2) that they almost never cite Anglo-American journals, it is dubious that they are following standard citation practices.In order to prove this, it would have to be studied more systematically. Our figures constitute a tempting first step in this direction.

Finally, drawing a global picture (a long-term goal, in terms of database construction) would require to integrate journals from other places, especially from Germany and South America.

### Explanatory considerations: a license to cite?

French citation practice can be traced to context. French psychoanalytic thought has always partly taken place outside of the academic world, thereby following rules different from standard academic discourse, for at least two reasons.

First, analysts appointed to full professorships in psychoanalytically-oriented psychology (D. Lagache, J. Laplanche) had already gained prominence in analytic schools. Second, becoming a licensed clinical psychologist in France doesn't require a full course of graduate study: it is a 5-year Masters-level training, which aims at training psychologists for clinical work in non-academic institutions. Thus, psychoanalytic thought taught in such curricula mostly feeds clinicians who are not very concerned with academic research.

This relative academic extraterritoriality of French psychoanalytic thought has an impact on its visibility. A shared legacy inherited by the next generations, it is mostly based upon the elaboration of local clinical practices: mental care institutions, supervision groups in psychoanalytic schools, and the training of licensed psychologists. In these contexts, this legacy is often already shared by clinicians; and when it isn't (as in universities, where students become acquainted with it), it is referred to in a practice-oriented, citation-free manner. Therefore, French psychoanalytic publications often refer to this shared legacy in an implicit manner, at odds with the explicit citation conventions that are standard practice in contemporary academy.

Perhaps, French psychoanalytic researchers have imposed restrictions upon themselves, inhibited about switching from their inherited, implicit mode of referring to an explicit mode of acknowledgment through citation^1^. They would, so to speak, lack the license to cite. This could account for the discrepancy between French and Anglo-American citations in French psychoanalytic journals (Table 2): the extreme scarcity of Anglo-American journal citations could be understood as the effect of an injunction to remain faithful to a national heritage.

## Conclusion

The current lack of international outreach should be viewed as an opportunity. French psychoanalysts should not be afraid to lose their singularity, as in the narcissism of minor differences (Freud, 1921, 1930; Gabbard, 1993): engage with fellow international researchers, which will help to clarify their clinical and theoretical originality.

However, such engagement would entail a precondition concerning research. Articles should be written not for peers who share an implicit network of references, but for an international community with diverse, local legacies, with the ultimate aim of promoting an exchange of perspectives. Aspiring for international exchanges requires that one takes recent work into greater account. While this focus might rely on the short timespan of algorithmic measures of citation rate, and therefore has questionable epistemic justification, it can also stir the field's vitality. Even though citation practices do differ among disciplines (as between, say, biology and psychoanalysis), the rate of citations of recent productions could count as an interesting proxy for field vitality. The experience of one of the authors (EJ) is telling: he is the editor of the psychoanalytic journal that has the highest impact factor over the last 5 years, and has found that the increase in impact factor, has affected both the quality and the quantity of submissions in a positive way^2^.

Finally, both French and English-speaking researchers and journals could benefit from an increase in citational acknowledgment, which could help unite the field around common, trans-national challenges. For example, an interesting—if complex—avenue for psychoanalytic researchers would be to try and expand bibliometric quality parameters. Since psychoanalytic research primarily aims at developing therapeutic theories and practices (a distinctive status within the social science), an interesting task would be to try and devise a social value metrics, reflecting the social value of psychoanalytic research. Such a goal, which might well complement the proliferating research on the assessment of therapeutic change, would certainly require an international collaboration.

## Author contributions

RP and OP are equal contributors to this paper (co-first authors). RP has put together the original database on the basis of Scopus, and has calculated the results. He has contributed to writing the paper (structure of the argument); he has co-constructed the interpretation of the results and of their limits, and their discussion. OP has contributed to the structure of the argument, and has written the detailed version of the paper. He has co-constructed the interpretation of the results and of their limits, and their discussion. CD has contributed to the design of the original database, to calculating the results and to their statistical interpretation. EJ has contributed to writing the paper (structure of the argument, references). All authors read and approved the final manuscript.

### Conflict of interest statement

The authors declare that the research was conducted in the absence of any commercial or financial relationships that could be construed as a potential conflict of interest.

## Footnotes

^1^This contrast with academic citation conventions has become much stronger since the digital globalization of research, which entails: 1) a new visibility – abstracts can be freely consulted from anywhere, often just by clicking on a database hyperlink (cf. e.g. Pubmed, and the “cited by” function in Google scholar or PEP); and 2) a quicker pace: the rapid availability of research productions leads to a focus on currently debated topics and publications, which attract most citations.

^2^Also, not all algorithms make room for the specificity of clinical journals, which don't cite as much as empirical research-oriented journals – partly because of the complex relationship between data and their interpretation. While JCR's Impact Factor increases this discrepancy in favor of empirical research journals by comparing the average raw number of cites per paper, SCImago's SJR division of psychology into sub-specialties makes it possible to even things out, by limiting relative journal citedness to sub-fields. This protects the specificity of clinical work. As an example, well-esteemed clinical journals such as *Psychoanalytic Psychology* (PP) and the *International Journal of Psycho-Analysis* (IJPA) both belong to SCImago's 2^nd^ best quartile, Q2; so does, in the sub-field of cognitive & experimental psychology, a journal like *Emotion Review*. But in terms of Impact Factor, the clinical journals respectively reach 0,83 (PP) and 0,72 (IJPA), while *Emotion Review* reaches 4,73 – a 1 to 5 ratio.
